# Sea-level projections representing the deeply uncertain contribution of the West Antarctic ice sheet

**DOI:** 10.1038/s41598-017-04134-5

**Published:** 2017-06-20

**Authors:** Alexander M. R. Bakker, Tony E. Wong, Kelsey L. Ruckert, Klaus Keller

**Affiliations:** 1 Earth and Environmental Systems Institute, Pennsylvania State University, University Park, Pennsylvania, PA 16802 USA; 2 Rijkswaterstaat, Ministry of Infrastructure and Environment, Utrecht, Netherlands; 3 Department of Geosciences, Pennsylvania State University, University Park, Pennsylvania, PA 16802 USA; 4 Department of Engineering and Public Policy, Carnegie Mellon University, Pittsburgh, PA 15289 USA

## Abstract

There is a growing awareness that uncertainties surrounding future sea-level projections may be much larger than typically perceived. Recently published projections appear widely divergent and highly sensitive to non-trivial model choices_._ Moreover, the West Antarctic ice sheet (WAIS) may be much less stable than previous believed, enabling a rapid disintegration. Here, we present a set of probabilistic sea-level projections that approximates the deeply uncertain WAIS contributions. The projections aim to inform robust decisions by clarifying the sensitivity to non-trivial or controversial assumptions. We show that the deeply uncertain WAIS contribution can dominate other uncertainties within decades. These deep uncertainties call for the development of robust adaptive strategies. These decision-making needs, in turn, require mission-oriented basic science, for example about potential signposts and the maximum rate of WAIS-induced sea-level changes.

## Introduction

Future sea-level rise poses nontrivial risks for many coastal communities^1, 2^. Managing these risks often relies on consensus projections like those provided by the IPCC^3^. Yet, there is a growing awareness that the surrounding uncertainties may be much larger than typically perceived^4^. Recently published sea-level projections appear widely divergent and highly sensitive to non-trivial model choices^4^. Moreover, the West Antarctic ice sheet (WAIS) may be much less stable than previously believed, enabling a rapid disintegration^5, 6^. In response, some agencies have already announced to update their projections accordingly^7, 8^.

The construction of sea-level projections is often largely motivated by scientific considerations, such as gaining a better understanding of the underlying physics^2, 9^. In this process, the translation from input data to model projections and full uncertainty estimates involves a wide range of non-trivial model choices and assumptions that can result in large discrepancies between different uncertainty estimates^4^. For example, many studies consider a high level of model detail indispensable for reliable projections^3^, whereas semi-empirical modeling approaches^10–12^ trade complexity for the ability to calibrate the model. Semi-empirical modeling approaches often rely on strong assumptions about the prior parameter distributions, what mechanisms to include, and how to interpret and represent the data-model discrepancies. These modeling choices can be nontrivial and the associated uncertainties hard to quantify^13^. On the other hand, projections based on multi-model ensembles (implicitly) focus on structural uncertainty which requires strong assumptions on which part of the overall uncertainty is covered^4^.

Decision makers often prefer “robust” over optimal decisions when faced with “deep” uncertainty^14–18^. Deep uncertainty refers to a situation when experts cannot agree upon or are not willing to provide probabilistic uncertainty ranges^15^. In the context of decision-making, robustness has many different definitions that usually involve trading some optimality for relative insensitivity to deviations from the model assumptions or relatively good performance over a wide range of futures^15–18^.

Here we present sea-level projections to inform the design of robust strategies to cope with the deep uncertainties surrounding sea-level change, i.e. “solutions capable of withstanding from deviations of the conditions for which they are designed”^17^. This notion of “robustness” deviates from *scientific* robustness that builds on arguably well understood physics and empirical/robust evidence^19–21^, which may lead to overconfident uncertainty ranges^2, 4^ and getting surprised by new insights and data^9^.

Our sea-level projections are constructed to support robust decision frameworks by i) being explicit about the relevant uncertainties, both shallow and deep; ii) communicating plausible ranges of sea-level rise, including the deep uncertainties surrounding future climate forcings and potential WAIS collapse; and iii) tending to err on the side of underconfident versus overconfident when possible.

### Model design

We design the projections to be probabilistic where reasonable and explicit about deep uncertainties (e.g. resulting from non-trivial model choices) when needed. Robust decision frameworks often apply plausible rather than probabilistic ranges to represent and communicate uncertainties^17, 22^. In the case of sea-level projections, the bounding of the plausible range usually involves both a probabilistic interpretation of the surrounding uncertainties and estimates of which probabilities are still relevant. For example, a full disintegration of the major ice sheets is often not taken into account because the probabilities of this occurring are considered too small to be relevant^3, 23^. What probability is relevant is highly dependent on the decision context and therefore it makes sense to be explicit about the probabilities. Moreover, probabilities are the easiest and most unambiguous way to communicate uncertainties^24, 25^.

Our projections are designed to highlight the relatively large deep uncertainties, notably those resulting from future climate forcings and those surrounding potential WAIS collapse (even though representations of deep uncertainty often implicitly encompass probabilistic interpretations). The future climate forcing is, to a large extent, controlled by future human decisions.

The probability of a WAIS collapse is potentially much larger than previously thought due to the combined effects of Marine Ice Sheet Instability (MISI), ice cliff failure and hydrofracturing^5, 6^. The discovery of this new mechanism puts earlier expert elicitations in a different light as it is unclear if those were based on this combined effect. One approach when faced with deeply uncertain model structures and priors is to present a potential WAIS collapse as deeply uncertain by means of a plausible range. We stress that this range is not meant to represent an implicit probabilistic projection of the WAIS contribution to sea-level rise.

We merge some small deep uncertainties into the probabilistic part of the projections. According to Herman *et al*.^17^ “… a larger risk lies in sampling too narrow a range (thus ignoring potentially important vulnerabilities) rather than too wide a range which, at worst, will sample extreme states of the world in which all alternatives fail”. Thus, in the context of informing robust decision making, it can be preferable to be slightly under- than slightly overconfident. To minimize the risk of producing overconfident projections we only use observational data with relatively uncontroversial and well-defined error structure.

### Model setup

We use a relatively simple (39 free physical and statistical parameters), but a mechanistically motivated model framework to link transient sea-level rise to radiative concentration pathways applying sub-models for the global climate, thermal expansion (TE), and contributions of the Antarctic ice sheet (AIS), Greenland ice sheet (GIS) and glaciers and small ice caps (GSIC) (see Methods). This approach extends on the semi-empirical model setup recently reported by Mengel *et al*.^12^.

**Figure 1:**
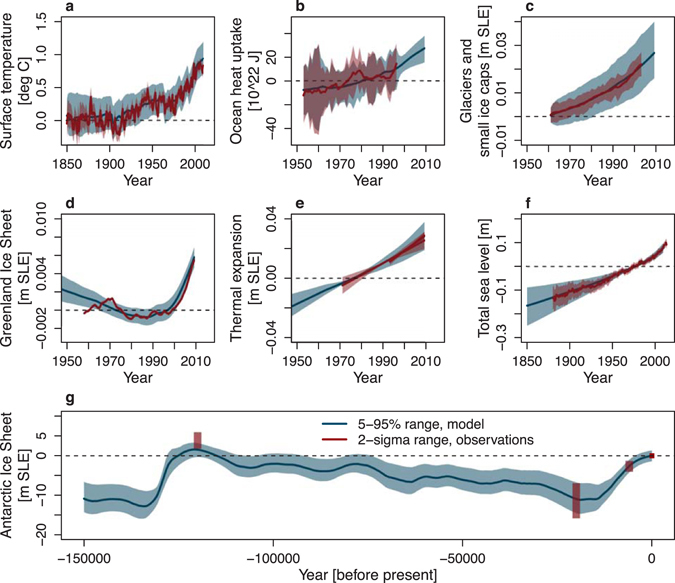
Past observations (red) and hindcasts (blue) global temperature, ocean heat content, sea-level contribution and global sea-level. Shadings represent the uncertainty (2σ) in the observational data and the 5–95% range in calibrated hindcasts.

We use a Bayesian calibration method, wherein paleoclimatic data is assimilated with the AIS model separately from the calibration for the rest of the model, which assimilates only modern observations. Modern model simulations are then run at parameters drawn from the two resulting calibrated parameter sets (AIS and rest-of-model) and compared to global mean sea-level (GMSL) data^26^ (see Methods). Only model realizations which agree with each GMSL data point to within 4σ are admitted into the final ensemble for analysis. 4σ was chosen so the spread in the model ensemble characterizes well the uncertainty in the GMSL data (Fig. 1f).

We choose, at this time, not to use paleo-reconstructions nor reanalyses, beyond incorporating a windowing approach into our calibration method for the Antarctic ice-sheet parameters. This choice is motivated by the highly complex and uncertain error structure of these data sets. Failure to account for such complex error structure can result in considerable overconfidence, especially for low-probability events^27^.

### Observational data and hindcasts

Global temperature, ocean heat, and most sea-level contributions have typically been subject to upward, slightly accelerating trends since 1850 (Fig. 1)^3^. Only the sea-level contribution from the AIS has been close to zero and might even have been slightly negative^3^. The reliability of the datasets decreases back in time due to the lower data availability and only the datasets for global mean surface temperature and global mean sea level go back to before 1950.

For the oceanic thermal expansion we use trends (together with the uncertainty estimates) as reported by the IPCC^3^ for the calibration (Table S1). The time scale and uncertainties of the paleoclimatic AIS data are substantially different from those of the observational data for other components of sea-level rise. For this reason, we calibrate the AIS model separately from the others, based on paleo-data as previously used by Shaffer^28^ and Ruckert *et al*.^29^ (see Methods).

Note that the calibrated model performance depends on the assumed statistical model, the observational data, and the adopted physical model structure. We have purposefully implemented a modular modeling framework that can be easily modified to incorporate new observational data and model structure; this framework will be the subject of a follow-up study.

In general, the calibrated hindcasts (including both statistical and parameter uncertainty) correspond reasonably well to the reported uncertainty ranges surrounding the observational data. After calibration *and post-calibration* our hindcasts of especially global temperature and global sea-level match the observations fairly well whereas the component models show some small deviations; the high AIS contribution during the Last Interglacial period is somewhat underestimated and GSIC uncertainty is slightly too large. However, the latter matches our premise that it is better to be slightly underconfident than overconfident.

### Projections

**Figure 2:**
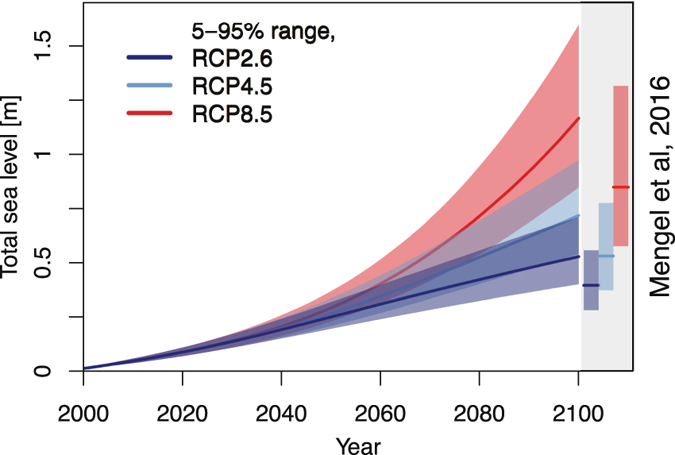
Future probabilistic global sea-level projections for the 21^st^ century under RCP2.6 (dark blue), RCP4.5 (light blue) and RCP8.5 (red) forcing scenarios^30^, compared to the projections for 2100 by Mengel *et al*.^12^ (vertical side bars).

**Figure 3:**
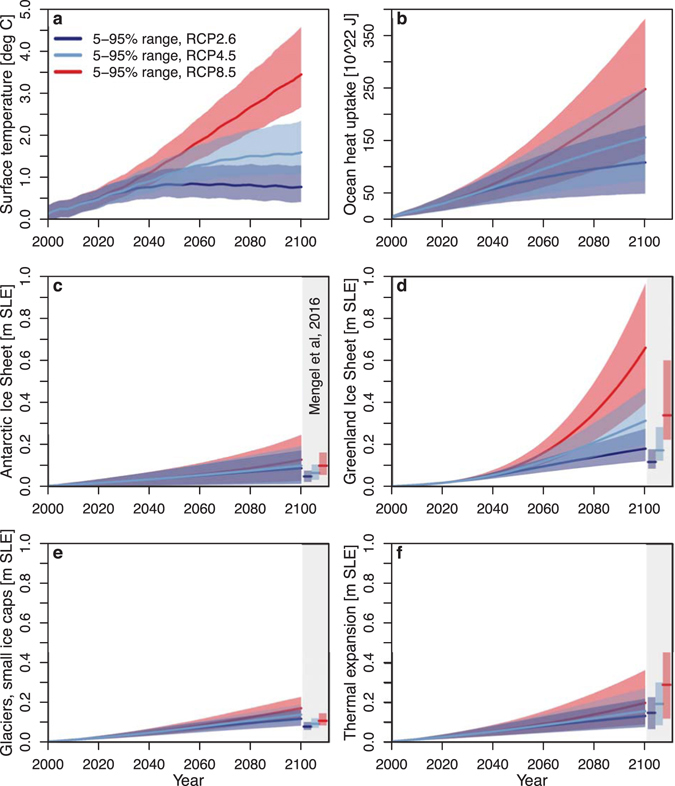
Future probabilistic projections of global temperature, ocean heat content and sea-level contributions.

For the first decades, the choice of RCP hardly affects the overall uncertainty (Fig. 2). The probabilistic part of the sea-level projections for 2050 yields 90% credible ranges of 0.19–0.32 m (RCP2.6), 0.21–0.34 m (RCP4.5), and 0.23–0.37 m (RCP8.5) sea level relative to 1986–2005 mean sea level. These ranges are largely shaped by the uncertain contributions of the AIS and thermal expansion (Fig. 3).

Around 2040–2050, a large and uncertain contribution of the GIS becomes important, of which the amount is highly dependent on the RCP (Fig. 3). This GIS contribution increases the 90% credible ranges for 2100 to 0.40–0.71 m (RCP2.6), 0.54–0.97 m (RCP4.5), and 0.85–1.59 m (RCP8.5) sea level in 2100, relative to 1986–2005 mean sea level (Fig. 2). This is slightly higher than projected by the recent and comparable study of Mengel *et al*.^12^ (that projects 0.28–0.56 m, 0.37–0.77 m, and 0.57–1.31 m for RCP2.6, RCP4.5, and RCP8.5, respectively) and can be explained by the relatively large contributions from the large ice bodies (Fig. 3 and Table S2). Our projected 5–95% uncertainty ranges in global sea-level are however quite similar to the results of Mengel *et al*.^12^.

This similarity seems somewhat surprising since we deliberately aimed to be conservative with our prior parameter choices, but can be explained by our two-step calibration approach (see Methods). In the first step we calibrate the individual components of sea-level rise separately (similarly to Mengel *et al*.^12^), which indeed gives much wider uncertainty ranges in the projected sea-level rise and its components (not shown). Yet, those separate ranges are considerably reduced by the second combined calibration step that also assimilates global sea-level data.

### Deep uncertainties

Pollard *et al*.^5^ suggests that a WAIS collapse is possible on the order of decades. Yet, the timing of a rapid disintegration is deeply uncertain. DeConto and Pollard^6^ present four widely divergent uncertainty ranges with, depending on the model choices, central estimates ranging from 64 to 114 cm for the sea-level contribution at 2100 following RCP8.5.

**Figure 4:**
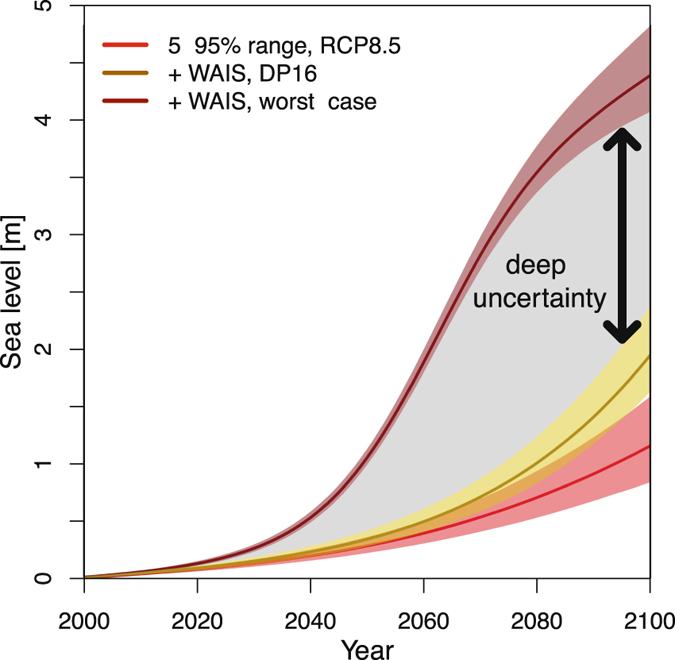
Future sea-level projections including deeply uncertain contribution of the WAIS.

Our study contributes to communicating this deep uncertainty in the cumulative contribution of the WAIS by characterizing the effect on plausible changes in global sea-level rise given the additional processes such as oceanic thermal expansion. We provide three projections based on three WAIS-collapse scenarios, following RCP8.5; no collapse (0 cm), a mid-range estimate (79 cm in 2100, based on DeConto and Pollard^6^, and a high case (3.3 m, full WAIS disintegration within a couple decades^5^) (Fig. 4). For 2100, this implies a factor of two to four wider uncertainty range. For the period prior to 2100, this factor could be even larger. This deep uncertainty is a potentially important input to the design of robust strategies to cope with the sea-level response to anthropogenic climate change. It is important to note that we do not intend to assign an implicit probability distribution to these deeply uncertain projections. We simply want to characterize and communicate key aspects of the deeply uncertain WAIS contribution to sea-level rise.

## Conclusions and Discussion

We presented a set of sea-level projections designed to represent important deep uncertainties and to inform robust decision-making frameworks. Our simple model framework includes semi-empirical models of the climate and sea-level contributions from thermal expansion, the Antarctic ice sheet, the Greenland ice sheet, and glaciers and small ice caps. Its relative simplicity is chosen to result in a transparent model structure and to enable a data-model fusion. Our calibration is designed to avoid overconstraining the projections. We hence only utilize observational data accompanied with clear uncertainty estimates, and aim for relatively non-informative prior distributions. We communicate divergent expert assessments and large structural uncertainties as deep uncertainties surrounding the projections.

The deeply uncertain contribution of WAIS disintegration dominates the overall uncertainty surrounding the sea-level projections within decades. We present examples of low and high sea-level rise scenarios that could be expanded by relying more heavily on expert elicitation^31, 32^ or by incorporating strong priors on the characterization of the West Antarctic deep uncertainties.

## Methods

### Semi-empirical model framework

We combine previously published, semi-empirical models (Table S1). The global temperature and ocean heat content are simulated with the coupled zero dimensional climate and 1D ocean model DOECLIM^33^. The global mean surface temperature anomaly (*T*_*g*_) feeds into the four models of sea-level contribution from thermal expansion^12^, glaciers and small ice caps (submodel of MAGICC)^34^, the Greenland ice sheet (SIMPLE)^35^, and the Antarctic ice sheet (DAIS)^28^.

The DAIS model also requires Antarctic ocean surface temperatures (*T*_*ANTO*_) which we estimate from a simple linear relation with *T*_*g*_ bounded below at the freezing point of salt water (*T*_*f*_ = −1.4 °C),

1 $${{\rm{T}}}_{ANTO}={{\rm{T}}}_{f}+\frac{{{\rm{a}}}_{ANTO}\ast {T}_{g}+{{\rm{b}}}_{ANTO}-{{\rm{T}}}_{f}}{1+\exp [({{\rm{a}}}_{ANTO}{\ast T}_{g}+{{\rm{b}}}_{ANTO}-{{\rm{T}}}_{f}){/a}_{ANTO}]}$$

where *a*_*ANTO*_ is the sensitivity of the Antarctic ocean temperature to global mean surface temperature (unitless), and *b*_*ANTO*_ is the Antarctic ocean temperature for *T*_*g*_ = 0 °C. *a*_*ANTO*_ and *b*_*ANTO*_ are both estimated as uncertain model parameters.

For the models with four or fewer physical parameters (thermal expansion (TE)-model, MAGICC-GSIC, SIMPLE, and ANTO) we calibrate all parameters. For DOECLIM we apply the same free (physical) parameters as Urban *et al*.^36^ (climate sensitivity (*S*), the aerosol amplification factor (*α*), and the ocean vertical diffusivity (*κ*)), and for DAIS the same as used by Shaffer^28^ and Ruckert *et al*.^29^.

### Model calibration

The model calibration approach consists of two stages. In the first stage, the AIS model is calibrated using paleoclimatic data as in Ruckert *et al*.^29^, along with trends in the AIS mass balance from the IPCC AR5^3^. The rest of the model components are similarly calibrated using modern observations. The reason for the separate calibrations is the vastly different temporal scale and characterization of errors between the paleoclimatic versus the modern data. All of the calibration data are detailed in Table S1. The model calibration is done using a robust adaptive Markov chain Monte Carlo (MCMC) approach^37^. For both the paleoclimatic and the modern calibrations, Gelman and Rubin diagnostics are examined to assess convergence^38^.

All parameters are assigned wide, physically-motivated prior ranges (Table S3), intentionally taken at least as wide as ranges considered in previous studies^29, 36^ or divergent estimates from the literature (Table S4). We rely on published ranges, if these ranges are derived from data other than we use for the full calibration. For example, climate sensitivity is one of the parameters of our climate model, but published uncertainty ranges rely often on the same past observational data. Using those uncertainty ranges as prior would double-count the information content in the data. If independent priors are not available, we formulate priors that are constrained by our mechanistic understanding, and pre-calibration^39^. This approach is one potential source of deep uncertainty, especially in case of limited availability of data to update the prior distribution. We are not aware of uncontroversial prior distributions for a potential rapid ice sheet contribution of the West Antarctic ice sheet and we therefore restrict ourselves to a deeply uncertain range.

In the paleoclimatic calibration, four parallel MCMC chains of 500,000 DAIS model realizations each are sampled. The first 120,000 iterations of each is removed for burn-in, yielding 1,520,000 posterior parameter estimates for analysis. For the modern calibration, four parallel MCMC chains of 1,000,000 iterations each of the coupled DOECLIM-thermal expansion-GSIC-GIS model (modern calibration) are simulated. The last 500,000 iterations from each chain are used for analysis as the calibrated “rest-of-model” parameter estimates, yielding 2,000,000 posterior parameter samples for analysis.

50,000 sample parameter sets are drawn from the DAIS and rest-of-model calibrated parameter sets. The entire parameter combination at which the models were run is preserved in this sampling. What is lacking at this stage is the joint rest-of-model and DAIS parameter distribution. The post-calibration step estimates this link by running the entire BRICK sea-level rise module (DOECLIM-ANTO-thermal expansion-GSIC-GIS-AIS) at these sampled parameter values. The parameter combinations are restricted to only those which yielded model realizations for global mean sea-level (GMSL) which matched data^26^ to within a four-sigma window around all GMSL data points. The four-sigma range was chosen so as not to overconstrain, but still restrict the ensemble to simulations with a realistic representation of GMSL. Out of the 50,000 posterior samples, 5,612 post-calibrated model simulations are found. These served as the parameter samples for projections of GMSL. Projections to 2100 of GMSL and its components (thermal expansion, GSIC, GIS, and AIS) are made using Representative Concentration Pathways 2.6, 4.5, and 8.5^30^. Experiments conducted using alternative windowing approaches for the GMSL post-calibration show little (at most five centimeters) variation in the 5–95% ranges of projected sea-level rise by 2100.

### Data availability

The sea-level rise model (consisting of the subcomponents of sea-level rise used here, are available at https://github.com/scrim-network/BRICK/tree/robustslr. Large parameter files and model results files are available from https://download.scrim.psu.edu/Wong_etal_BRICK.

## Electronic supplementary material

SUPPLEMENTARY INFORMATION
