# Supplementary material for: Sea-level projections representing the deeply uncertain contribution of the West Antarctic ice sheet
Source: Sci Rep. 2017 Jun 20;7:3880. doi: 10.1038/s41598-017-04134-5 (PMC5478629; doi:10.1038/s41598-017-04134-5)
Supplement: Supplementary file 1 — SUPPLEMENTARY INFORMATION [file 41598_2017_4134_MOESM1_ESM.pdf]

SUPPLEMENTARY INFORMATION

Sea-level projections representing deeply uncertain ice-sheet contributions

Alexander M.R. Bakker[1,2,*], Tony E. Wong[1], Kelsey L. Ruckert[1], and Klaus Keller[1,3,4]

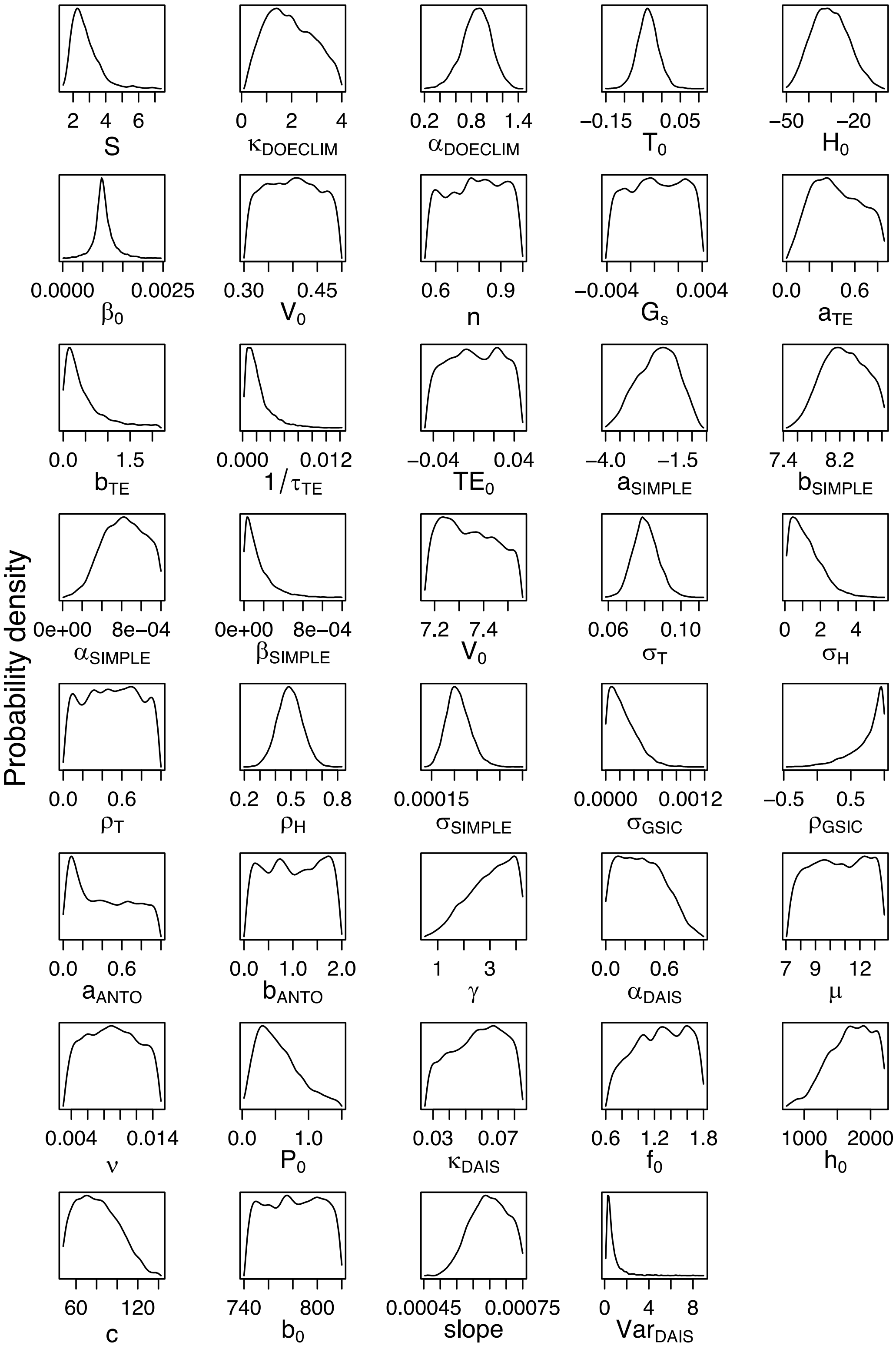

Figure S1 | Marginal posterior distributions of 39 free parameters.

Contribution	Model	Data
Surface temperature	DOECLIM (Kriegler, 2005)	Morice et al (2012?),
Ocean heat uptake	DOECLIM (Kriegler, 2005)	Gouretski and Koltermann, 2007
Glaciers and small ice caps	GSIC-MAGICC (Wigley and Raper, 2005)	Dyurgerov and Meier, 2005
Thermal expansion	TE (Mengel et al, 2016)	Church et al. 2013
Greenland ice sheet	SIMPLE (Bakker et al, 2016)	Sasgen et al. 2012
Antarctic ice sheet	DAIS (Shaffer 2014)	Shaffer 2014, Ruckert 2016, Church et al. 2013

Table S1 | Component models and calibration data used.

Contribution	RCP2.6	RCP4.5	RCP8.5
Thermal expansion	13.3 (7.65-22.0)	15.8 (8.24-27.4)	19.9 (9.00-36.5)
Glaciers and small ice caps	11.9 (8.34-16.5)	14.0 (9.84-19.1)	17.2 (12.3-22.9)
Greenland ice sheet	18.1 (12.1-27.5)	31.3 (20.2-47.2)	66.1 (39.8-96.9)
Antarctic ice sheet	8.73 (1.25-17.2)	9.97 (1.79-19.4)	12.8 (2.78-24.7)
Total	52.6 (39.9-70.8)	71.5 (53.9-97.1)	116 (84.5-159)

Table S2 | Sea-level contribution in 2100, relative to 1986-2005 mean sea level (cm SLE)

Parameter	Model	Median	5%	95%	Lower	Upper	Units
S	DOECLIM	2.552	1.787	4.317	0.1	10	°C
$\kappa_{DOECLIM}$	DOECLIM	1.888	0.4791	3.656	0.1	4	$cm^2\ s^{-1}$
$\alpha_{DOECLIM}$	DOECLIM	0.8759	0.553	1.158	0	2	-
T_0	DOECLIM	-0.03695	-0.08114	0.01047	-0.3	0.3	°C
H_0	DOECLIM	-30.89	-43.15	-16.72	-50	0	10^{22} J
σ_T	DOECLIM	0.08029	0.06989	0.09215	0.05	5	°C
σ_H	DOECLIM	1.054	0.1891	2.835	0.1	10	10^{22} J
ρ_T	DOECLIM	0.5005	0.04763	0.9465	0	0.999	-
ρ_H	DOECLIM	0.4915	0.3657	0.6244	0	0.999	-
β_0	GSIC	0.000997	0.00066	0.001464	0	0.041	$m\ yr^{-1}$
$V_{0,GSIC}$	GSIC	0.3997	0.3103	0.4896	0.3	0.5	m
n	GSIC	0.78	0.5738	0.9773	0.55	1	-
$G_{s,0}$	GSIC	4.45E-05	-0.003625	0.003688	-0.0041	0.0041	m
σ_{GSIC}	GSIC	0.0002078	1.81E-05	0.0006155	0	0.0015	m
ρ_{GSIC}	GSIC	0.8424	0.2056	0.9891	-0.999	0.999	-
a_{TE}	TE	0.4265	0.1051	0.8074	0	0.8595	$m\ °C^{-1}$
b_{TE}	TE	0.33	0.0337	1.728	0	2.193	m
$1/\tau_{TE}$	TE	0.00172	0.0004294	0.005997	0	1	yr^{-1}
TE_0	TE	0.0005103	-0.04287	0.04339	-0.0484	0.0484	m
a_{SIMPLE}	SIMPLE	-2.142	-3.364	-1.121	-4	-0.001	$m\ °C^{-1}$
b_{SIMPLE}	SIMPLE	8.255	7.766	8.745	5.888	8.832	m
α_{SIMPLE}	SIMPLE	0.0006293	0.0002762	0.0009549	0	0.001	$yr^{-1}\ °C^{-1}$
β_{SIMPLE}	SIMPLE	0.0001002	7.71E-06	0.0004376	0	0.001	yr^{-1}
$V_{0,SIMPLE}$	SIMPLE	7.347	7.178	7.54	7.16	7.56	m
σ_{SIMPLE}	SIMPLE	0.0002067	0.0001697	0.0002544	0	0.002	m
ρ_{SIMPLE}	SIMPLE	0.8821	0.8821	0.8821	0.8821	0.8821	-
a_{ANTO}	ANTO	0.4399	0.03937	0.9479	0	1	$°C\ °C^{-1}$
b_{ANTO}	ANTO	1.005	0.1052	1.896	0	2	°C
γ	DAIS	3.113	1.391	4.146	0.5	4.25	-
α_{DAIS}	DAIS	0.3611	0.03808	0.7807	0	1	-
μ	DAIS	10.46	7.432	13.38	7.05	13.65	$m^{1/2}$
ν	DAIS	0.008997	0.003788	0.01429	0.003	0.015	$m^{-1/2}$
P_0	DAIS	0.5062	0.1276	1.253	0.026	1.5	$m\ yr^{-1}$
κ_{DAIS}	DAIS	0.05742	0.02845	0.08192	0.025	0.085	$°C^{-1}$
f_0	DAIS	1.264	0.6927	1.744	0.6	1.8	$m\ yr^{-1}$
h_0	DAIS	1718	1094	2161	735.5	2206.5	m
c	DAIS	80.05	51.2	120.9	47.5	142.5	$m\ °C^{-1}$
b_0	DAIS	780.4	744.3	816.6	740	820	m
slope	DAIS	0.0006488	0.0005511	0.0007352	0.00045	0.00075	-
Var_{DAIS}	DAIS	0.5067	0.1887	2.262	0	2	m^2

Table S3 | Parameters, units, models, prior ranges, and posterior distribution statistics.

**Study**	**DOECLIM**	**MAGICC**	**SIMPLE**	**DAIS-ANTO**	**TE**
Urban & Keller (2010)	X				
Wigley & Raper (2005)		X			
Church et al (2001)		X			
Chen & Ohmura (1990)		X			
Marčelja (2010),					X
Mengel et al (2016)					X
Church & White (2011)		X	X	X	X
Bakker et al (2016)			X		
Ridley et al (2010)			X		
Bamber et al (2013)			X		
Applegate et al (2012)			X		
Robinson et al (2012)			X		
Shaffer (2014)				X	
Oerlemans (2005)				X	
Oerlemans (2003)				X	
Ruckert et al (2016)				X	
Kotlyakov (1966)				X	
Bryazgin (1986)				X	
Pollard & DeConto (2009)				X	
Thadathil et al (2002)				X	

Table S4 | Literature used to formulate prior probability density functions for the calibration

References

Applegate, P. J. *et al*. An assessment of key model parametric uncertainties in projections of Greenland Ice Sheet behavior. *The Cryosphere* 6, 589-606 (2012)

Bakker, A. M. R., Applegate, P. J. & Keller, K. A simple, physically motivated model of sea-level contributions from the Greenland ice sheet in response to temperature changes. *Environmental Modelling & Software* 83, 27-35 (2016)

Bamber *et al* A new bed elevation dataset for Greenland. *The Cryosphere* 7, 499-510 (2013).

Bryazgin (1986, "Method of preparing monthly charts of atmospheric precipitation in Antarctica")

Chen and Ohmura (1990, "Estimation of Alpine glacier water resources and their change since the 1870s")

Church, J. A. & White, N. J. Sea-level rise from the late 19th to the early 21st century. *Surveys in Geophysics* 32, 585–602 (2011).

Church, J. A. *et al.* Sea level change. In Stocker, T. F. et al. (eds.) *Climate Change 2013: The Physical Science Basis. Contribution of Working Group I to the Fifth Assessment Report of the Intergovernmental Panel on Climate Change*, 1137–1216 (Cambridge University Press, Cambridge, United Kingdom and New York, NY, USA, 2013).

Dyurgerov, M. B. & Meier, M. F. *Glaciers and the changing Earth system: a 2004 snapshot*. 58, (Institute of Arctic and Alpine Research, University of Colorado Boulder, 2005)

Gouretski, V. & Koltermann, K. P. How much is the ocean really warming? *Geophysical Research Letters* 34 (2007)

Kotlyakov (1966, "The snow cover of the Antarctic and its role in the present-day glaciation of the continent")

Kriegler, E. *Imprecise Probability Analysis for Integrated Assessment of Climate Change*. Ph.D. thesis, Universität Potsdam, Potsdam (2005). URL https://www.pik-potsdam.de/members/edenh/theses/PhDKriegler.pdf

Marčelja, S. The timescale and extent of thermal expansion of the global ocean due to climate change. *Ocean Science* 6, 179-184 (2010).

Mengel, M. *et al.* Future sea level rise constrained by observations and long-term commitment. *Proceedings of the National Academy of Sciences of the United States of America (PNAS)* 113, 2597–2602 (2016).Morice et al. (2012)

Oerlemans, J. Antarctic ice volume for the last 740 ka calculated with a simple ice sheet model. *Antarctic Science* 17, 281-287 (2005)

Oerlemans, J. A quasi-analytical ice-sheet model for climate studies. *Nonlinear processes in geophysics* 10, 441-452 (2003)

Pollard, D. & DeConto, R. M. Modelling West Antarctic ice sheet growth and collapse through the past five million years. *Nature* 458, 329-332 (2009)

Ridley, J. *et al.* Thresholds for irreversible decline of the Greenland ice sheet. *Climate Dynamics* 35, 1049-1057 (2010)

Robinson, A. *et al.* Multistability and critical thresholds of the Greenland ice sheet. *Nature Climate Change* 2, 429-432 (2012)

Ruckert, K. L., Shaffer, G., Pollard, D., Forest, C. E. & Keller, K. Neglecting cliff instability in Antarctic ice sheet models can reduce melting during warming periods. *PLoS ONE* (2016). In preparation.

Sasgen *et al.* Timing and origin of recent regional ice-mass loss in Greenland. *Earth and Planetary Science Letters* 333/334, 293-303 (2012).

Shaffer, G. Formulation, calibration and validation of the dais model (version 1), a simple antarctic ice sheet model sensitive to variations of sea level and ocean subsurface temperature. *Geoscientific Model Development* 7, 1803–1818 (2014).

Thadathil et al (2002, doi:10.1175/1520-0426-19.3.391).Ruckert, K. L., Shaffer, G., Pollard, D., Forest, C. E. & Keller, K. Neglecting cliff instability in Antarctic ice sheet models can reduce melting during warming periods. *PLoS ONE* (2016). In preparation.

Urban, N. M. & Keller, K. Probabilistic hindcasts and projections of the coupled climate, carbon cycle and Atlantic meridional overturning circulation system: a bayesian fusion of century-scale observations with a simple model. *Tellus* A 62, 737–750 (2010).

Wigley, T. M. L. & Raper, S. C. B. Extended scenarios for glacier melt due to anthropogenic forcing. *Geophysical Research Letters* 32 (2005)
